# Acute Effect of Electromyostimulation Superimposed on Running on Maximal Velocity, Metabolism, and Perceived Exertion

**DOI:** 10.3390/biology11040593

**Published:** 2022-04-14

**Authors:** Holger Stephan, Thorsten Hagedorn, Udo Frank Wehmeier, Fabian Tomschi, Thomas Hilberg

**Affiliations:** Department of Sports Medicine, University of Wuppertal, Moritzstraße 14, 42117 Wuppertal, Germany; hagedorn@uni-wuppertal.de (T.H.); wehmeier@uni-wuppertal.de (U.F.W.); tomschi@uni-wuppertal.de (F.T.); hilberg@uni-wuppertal.de (T.H.)

**Keywords:** electric stimulation, superimposed, exercise test, running, treadmill

## Abstract

**Simple Summary:**

Electromyostimulation is the activation of muscles via electrodes placed on the skin, often by wearing a special suit. The application can be passive or active to intensify the training. There are some studies on cycling with superimposed electromyostimulation. However, little is known about its use in running. Therefore, a group of young healthy men performed three treadmill tests in which speed was gradually increased until exhaustion. In one session they ran without electromyostimulation and in two other sessions with superimposed electromyostimulation. Metabolic response, exertion, and maximal performance were examined. Running with electromyostimulation resulted in a lower maximum running speed, was more strenuous, and, in some cases, more metabolically demanding than running without electromyostimulation. Superimposed electromyostimulation is feasible and intensifies running. Normal runners and those with extreme training volumes could benefit from its use.

**Abstract:**

Electromyostimulation has been shown to intensify exercise when superimposed on cycling. However, little is known about the application during running, which might help to prevent injuries linked to high running volumes, as intensification of running allows for a reduction in training volume. Therefore, the purpose of the study was to examine the effects of electromyostimulation superimposed on running. Men who were no younger than 18 and no older than 35 were eligible for inclusion in the study. Exclusion criteria were previous experience with electromyostimulation training, the presence of a contraindication according to the manufacturer, or a contraindication to physical activity. A sample of 22 healthy males with an ordinary performance capability accomplished three similar cardiopulmonary treadmill tests until exhaustion in a crossover study design that included lactate measurements and interrogations of perceived exertion. The first test was conducted without electromyostimulation and was followed in a randomized order by the second and the third test condition with 30 or 85 Hz stimulation, respectively, of the lower body. Superimposed electromyostimulation significantly reduced the maximal achieved velocity (control 15.6 ± 1.1 vs. 30 Hz 15.1 ± 1.2, *p* = 0.002; vs. 85 Hz 14.9 ± 1.1 km/h, *p* < 0.001), increased the perceived exertion at 10, 12 and 14 km/h (85 Hz + 0.7, *p* = 0.036; +0.9, *p* = 0.007; +1.3, *p* < 0.001; 30 Hz + 0.7, *p* = 0.025; +1.0, *p* = 0.002; +1.2, *p* < 0.001), and induced a significantly higher oxygen uptake at 8 km/h (85 Hz + 1.1, *p* = 0.006; 30 Hz + 0.9 mL·min^−1^·kg^−1^, *p* = 0.042), 10 km/h (30 Hz + 0.9 mL·min^−1^·kg^−1^, *p* = 0.032), and 14 km/h (85 Hz + 1.0 mL·min^−1^·kg^−1^, *p* = 0.011). Both electromyostimulation conditions significantly limited the maximal lactate level (30 Hz *p* = 0.046; 85 Hz *p* < 0.001) and 85 Hz also the recovery lactate level (*p* < 0.001). Superimposed electromyostimulation is feasible and intensifies running. Coaches and athletes could benefit from the increased training stimulus by reducing running velocity or volume, by combining endurance and strength training, and also by inducing better adaptations while maintaining the same velocity or volume. Therefore, electromyostimulation superimposed on running could be an interesting training tool for runners.

## 1. Introduction

Neuromuscular electrical stimulation (NMES) is an artificial approach to elicit contractions by current that causes a nonselective, synchronous, and spatially fixed recruitment of motor units [1]. It is not only used in rehabilitation settings but also in different healthy populations to maintain, restore, and improve muscle performance and muscular mass [2]. Both local and whole-body electromyostimulation (WB-EMS) devices are available, and differ in the stimulated muscle groups and application of electrodes. WB-EMS stimulates opponent working muscles concurrently with belts, whereas local EMS only stimulates separate muscle groups [3]. It can be assumed that sensory, motor, and sensorimotor regions of the brain are addressed by EMS [4]. Due to the possibility of addressing muscle chains given by WB-EMS, more functional exercises can be performed [3]. Electromyostimulation can be applied in different modi: (1) passively without voluntary contractions [5]; (2) superimposed on voluntary muscle work either with [6,7] or without additional weights [8,9]; and (3) combined with sessions without stimulation on different days [10]. In their systematic review, Pano-Rodriguez et al. [11] described the effects of WB-EMS under considering the risks of bias. The effects of WB-EMS on anthropometric characteristics are marginal. Blood parameters are not affected, there seems to be a benefit for psychophysiological parameters, and energy expenditure increased with WB-EMS. The main effects of WB-EMS are strength improvements, which have been frequently observed. However, there are also some high-standard deviations. Several studies investigated the effects of endurance-based cycling exercise with superimposed EMS [12,13,14,15,16,17,18]. The purpose of these studies was to consider if an augmented acute physiological response can be evoked via a simultaneous application of EMS. Solely Mathes et al. [18] focused on adaptations and chronic effects in addition to reactions to selected single units. Compared to regular cycling exercise, the following effects have been reported: a lower peak power output [12], a different hormonal balance [14,16], myokine balance [16,17], and exercise metabolism [12,14,15,18], including a higher oxygen uptake [13,15,18], a pronounced muscle damage [12,17,18], and perceived exertion [12,14], as well as an altered perceived physical state [14,17]. Several underlying mechanisms have been discussed. EMS puts more stress on muscles and resulted in more soreness and damage [12]. More pronounced damage has been demonstrated by superimposed stimulation, which involves more muscle mass and provokes additional eccentric muscle work [17]. Hormonal shifts are suggested to be owing to increased metabolic activity, subsidiary involvement of fast twitch fibers and motor units, higher amount of muscle damage [14], higher lactate levels [19], seen in Wahl et al. [14], fatigue [19], which is pronounced due to EMS [12], and pain [19], seen in another work by Wahl et al. [17]. The stimulation was applied either antagonistic (eccentric) or without consideration of the cycling rhythm. Most of the aforementioned studies used a constant workload. Furthermore, different stimulation protocols were applied, e.g., the impulse frequency ranged from 4 Hz to 85 Hz (partially frequency of 5000 Hz modulated at 40 Hz). However, the preference of single physical activities differs between regions. In some areas, running is preferred and running activities are more popular than cycling [20]. The relation between the amount of training distance covered by runners per week and injuries and also medical consultations must be considered and research shows that very high training volumes go along with an increased injury rate [21]. The aforementioned intensification of exercise caused by EMS allows a reduction in the training volume. For instance, WB-EMS can be considered as less time-consuming than the already efficient high intensity training [22]. Additionally, the hypothesized that a combination of aerobic and electrically evoked resistance training, by the application of antagonistic EMS during voluntary exercise [16], could be time saving when individuals are engaged in both fields. Therefore, a lower volume of injury incidence is conceivable when superimposed EMS is applied. However, there is a requirement for scientific research addressing running exercise with superimposed EMS and its potential positive effects on performance. A recent study applied a stimulation during 30-s running intervals. However, they only measured the long-term effects on performance [23]. The transferability of the data gathered on cycling with superimposed EMS to running with superimposed EMS is limited as there are several measurable differences between these physical activities. This emphasizes the importance of conducting studies for each, running and cycling, separately with respect to specificity [24]. Running potentially involves more muscle mass and is characterized by a higher delta efficiency, less compromised ventilation, and a distinct heart rate compared to cycling [25]. Furthermore, a pronounced involvement of fast twitch fibers during cycling has been suggested [26].

Therefore, the purpose of the study was to examine the effects of running with superimposed EMS on different performance and metabolism related parameters. The gained knowledge might be important for athlete runners with very high training volumes as EMS superimposed on running might reduce the risk of injury by at the same time maintaining or even increasing exercise intensity.

## 2. Materials and Methods

### 2.1. Participants

Men who were no younger than 18 and no older than 35 were eligible for inclusion in the study. Exclusion criteria were previous experience with electromyostimulation training, the presence of a contraindication according to the manufacturer (e.g., seizures, severe nephrological disease, severe neurological disorder), or a contraindication, especially according to the guidelines provided by Wonisch et al. [27], which refer to physical activity (e.g., acute carditis, acute coronary syndrome, febrile infections). Participants were recruited through public announcements and inquiries in the private sector. A total of 22 healthy males with an ordinary performance capability [28] were enrolled to participate in the study (Table 1). After the explanatory procedure, the participants signed a written consent to take part in the study. The trial was performed in accordance with the principles of the Declaration of Helsinki and approved by the ethics committee of the University of Wuppertal (MS/BBL 190124 Stephan).

### 2.2. Study Design and Procedures

**Figure 1:**
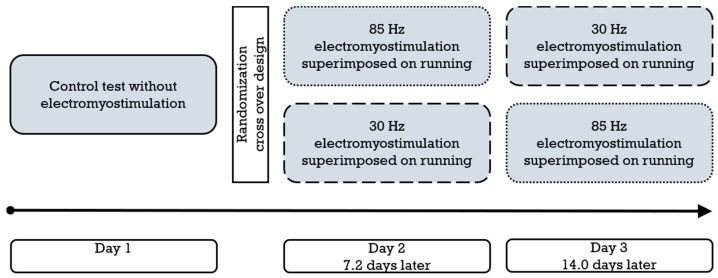
Schematic representation of the study design and procedure (*n* = 22). The dotted frame represents 85 Hz electromyostimulation, and the dashed frame represents 30 Hz electromyostimulation.

All participants were tested on three separate occasions owed to the crossover study design (Figure 1). In each session, a cardiopulmonary exercise test (spiroergometry) was conducted on a treadmill, which included lactate measurements and interrogations of perceived exertion. In addition to the medical checkup, a control intervention without stimulation took place on day 1, and supplementary superimposed stimulation with a randomly allocated impulse frequency of either 30 Hz or 85 Hz [12] was applied on day 2 (average 7.2 days later) and day 3 (average 14.0 days after the first test). Hence, both frequencies were only used once for each participant.

### 2.3. Medical Checkup

Participants underwent a medical checkup with recording of resting ECG, blood pressure, spirometry, and clinical examination to assess health status and suitability for cardiopulmonary exercise testing to exhaustion.

### 2.4. Spiroergometry, Lactate, and Perceived Exertion

The same standardized treadmill exercise testing protocol (XELG 70; Woodway, Waukesha, WI, USA) was used on all 3 examination days. During the entire test, the respiratory minute volume and exhaled gases were measured (JAEGER Vyntus CPX with SentrySuite software; CareFusion, Chicago, IL, USA) and an ECG was also administered (CAM-USB A/T Kiss with CardioSoft software; GE, Chicago, IL, USA). Before the exercise test started a baseline capillary blood sample was taken from participants in standing position from the right earlobe. During the subsequent 3 min treadmill familiarization period, participants maintained a predetermined velocity of 3.5 km/h at gradient of 1%. Subsequently, the incremental test started at 8 km/h with each phase lasting 3 min, followed by a resting phase of 30 s. During the rest periods, the treadmill was stopped, capillary blood samples were taken and the participants stated their perceived exertion based on the Borg scale. The velocity was increased by 2 km/h for each phase until the participants voluntarily stopped the trial by leaning on the handrail of the treadmill or giving a sign to the investigators. All participants were encouraged to run until exhaustion. The cool-down period lasted 10 min and comprised capillary blood sampling at 1, 3, 5, and 10 min. Lactate levels were ascertained from all capillary samples (Biosen S-Line Lab; EKF-diagnostic GmbH, Barleben, Germany). During all three cardiopulmonary exercise tests, participants wore a whole-body electrode suit, but superimposed electrostimulation occurred only on day 2 and day 3.

### 2.5. Electromyostimulation

On the second and third day, a supplementary superimposed stimulation with a randomly allocated impulse frequency of either 30 Hz or 85 Hz was applied (XBody Actiwave; XBody Hungary Kft., Győr, Hungary). Apart from the frequency, the stimulation pattern on day 2 and day 3 were equivalent (symmetric biphasic mode, rectangular shape of impulse, 400 µs impulse width, 10 s impulse interval duration, 5 s impulse pause, 1 s ramp-up). A stimulation intensity of 7 on a scale of 1 to 10 was set individually and for each stimulation session separately. Independently of the running rhythm, the musculature of the hip and the lower limbs (gluteals, quadriceps, hamstrings, and calves) were stimulated collectively.

### 2.6. Target Parameters

To evaluate the effect of electromyostimulation superimposed on running on metabolism, perceived exertion, and performance, oxygen uptake, respiratory exchange ratio, Borg scale score, and lactate level at the end of each stage, maximum running speed achieved, maximum oxygen uptake, maximum respiratory exchange ratio, maximum value selected on the Borg scale, maximum lactate level, and recovery lactate level were determined.

### 2.7. Statistical Analysis

The data are represented as mean ± standard deviation. Owing to the small sample sizes and the analysis of the normal distribution Wilcoxon test was applied to compare the results of the 3 single sessions with each other. The level of significance was set at *p* < 0.05 Effect sizes were calculated based on the results of the non-parametric tests (r ≥ 0.5 large effect; <0.5 to 0.3 medium effect; <0.3 to 0.1 small effect). The parameters extracted from the spiroergometric software (oxygen uptake and respiratory exchange ratio) representing the running stages were automatically averaged over 30 s. At each stage, peak oxygen uptake and the corresponding respiratory exchange ratio were determined and used for statistical analyses. Maximal oxygen uptake is represented by the highest value achieved using the same averaging technique, and maximal respiratory exchange ratio by the highest corresponding value achieved. If the last stage was terminated early, the achieved percentage of the scheduled time was multiplied by the velocity increment (2 km/h) and then added to the last accomplished velocity to calculate the maximal running velocity. In a few cases measurements were impaired for example due to a loosened breathing tube. Therefore, the data analysis could not always be carried out for all 22 included participants. Error resistant measurements (RPE, maximum velocity) could be conducted in 22 participants. Furthermore, one participant failed to finish 14 km/h.

## 3. Results

### 3.1. Feasibility

No participant reported any adverse effect of the EMS application on running movements. Further, no participant stopped the incremental running test due to the extra applied EMS. Above all, no running accident was observed during any running condition.

### 3.2. Stage Outcomes

**Figure 2:**
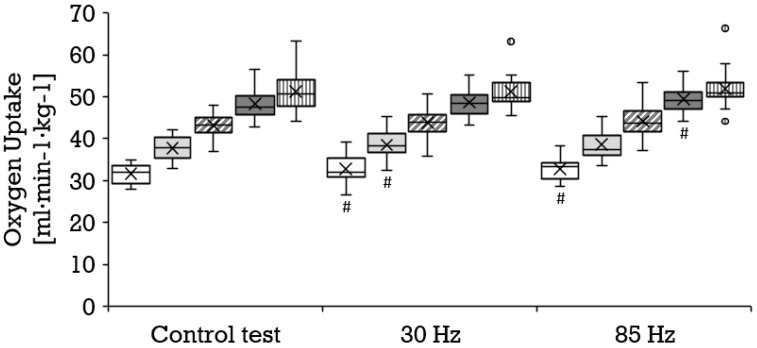
Distribution of the oxygen uptake (mL·min^−1^·kg^−1^) depicted as boxplot at the stages 8 km/h (white box, *n* = 17), 10 km/h (bright gray box, *n* = 20), 12 km/h (diagonally striped box, *n* = 20) and 14 km/h (dark gray box, *n* = 17), as well as for the maximum values (vertically striped box, *n* = 17) during the control test, running with 30 Hz and running with 85 Hz. Each filled circle is representing a discordant value. Significances (*p* < 0.05) compared to the control test are labeled with #.

**Figure 3:**
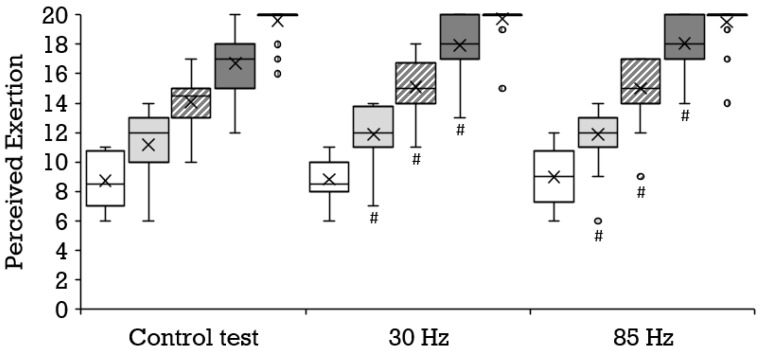
Distribution of the perceived exertion depicted as boxplot at the stages 8 km/h (white box, *n* = 22), 10 km/h (bright gray box, *n* = 22), 12 km/h (diagonally striped box, *n* = 22), and 14 km/h (dark gray box, *n* = 21), as well as for the maximum values (vertically striped box visible as black leveled line owed to the distribution, *n* = 22) during the control test, running with 30 Hz and running with 85 Hz. Each filled circle is representing a discordant value. Significances (*p* < 0.05) compared to the control test are labeled with #.

**Figure 4:**
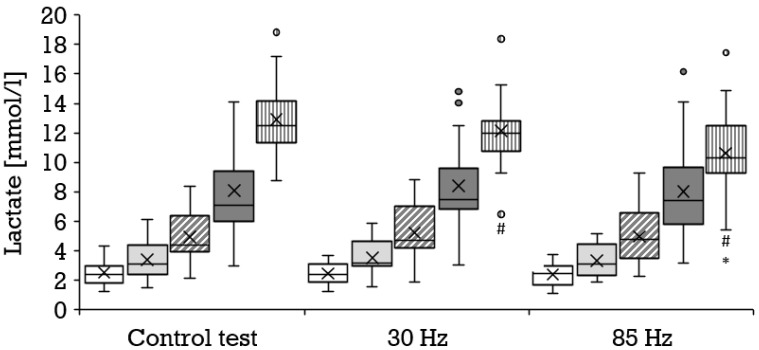
Distribution of the lactate values depicted as boxplot at the stages 8 km/h (white box, *n* = 21), 10 km/h (bright gray box, *n* = 21), 12 km/h (diagonally striped box, *n* = 21) and 14 km/h (dark gray box, *n* = 20), as well as for the maximum values (vertically striped box, *n* = 21) during the control test, running with 30 Hz and running with 85 Hz. Each filled circle is representing a discordant value. Significances (*p* < 0.05) compared to the control test are labeled with #, compared to 30 Hz with *.

Compared to the control condition, a significantly higher oxygen uptake was achieved with 85 Hz superimposed stimulation during 8 km/h (mean ± SD, 31.7 ± 2.3 vs. 32.8 ± 3.1 mL·min^−1^·kg^−1^; *p* = 0.006; r = 0.67) and 14 km/h (mean ± SD, 48.2 ± 3.5 vs. 49.2 ± 3.5 mL·min^−1^·kg^−1^; *p* = 0.011; r = 0.60); and with 30 Hz during 8 km/h (mean ± SD, 31.7 ± 2.2 vs. 32.6 ± 3.3 mL·min^−1^·kg^−1^; *p* = 0.042; r = 0.44) and 10 km/h (mean ± SD, 37.6 ± 2.7 vs. 38.5 ± 3.2 mL·min^−1^·kg^−1^; *p* = 0.032; r = 0.47). The results of the 85 Hz stimulation did not differ significantly from the results of the 30 Hz stimulation. Figure 2 illustrates the distribution and progression of the oxygen uptake during all three tests. During the control condition, a significantly lower respiratory exchange ratio was achieved than during running at 8 km/h with 85 Hz stimulation (mean ± SD, 0.88 ± 0.09 vs. 0.93 ± 0.06; *p* = 0.023; r = 0.55) and 30 Hz stimulation (mean ± SD, 0.88 ± 0.09 vs. 0.92 ± 0.07; *p* = 0.038; 0.45), as well as during running at 14 km/h with 85 Hz (mean ± SD, 1.10 ± 0.06 vs. 1.12 ± 0.06; *p* = 0.046; r = 0.47) and with 30 Hz (mean ± SD, 1.09 ± 0.06 vs. 1.12 ± 0.05; *p* = 0.009; r = 0.64). The selective consideration of tests with superimposed stimulation yielded no significant differences. With superimposed stimulation at 85 Hz, the perceived exertion during running was significantly greater than without additional stimulation at 10 km/h (mean ± SD, 11.2 ± 2.2 vs. 11.9 ± 2.0; *p* = 0.036; r = 0.45), 12 km/h (mean ± SD, 14.1 ± 1.8 vs. 15.0 ± 2.0; *p* = 0.007; r = 0.57) and 14 km/h (mean ± SD, 16.7 ± 2.1 vs. 18.0 ± 1.9; *p* = 0.000; r = 0.78). Likewise, 30 Hz elicited a significantly greater perceived exertion compared to the control test at 10 km/h (mean ± SD, 11.2 ± 2.2 vs. 11.9 ± 2.0; *p* = 0.025; r = 0.48), 12 km/h (mean ± SD, 14.1 ± 1.8 vs. 15.1 ± 2.0; *p* = 0.002; r = 0.66) and 14 km/h (mean ± SD, 16.7 ± 2.1 vs. 17.9 ± 2.1; *p* = 0.001; r = 0.73). The two different frequencies did not provoke significantly different responses. Figure 3 illustrates the distribution and progression of the perceived exertion during all three tests. The lactate values were similar at all stages in all tests (Figure 4).

### 3.3. Maximal Outcome and Recovery

**Figure 5:**
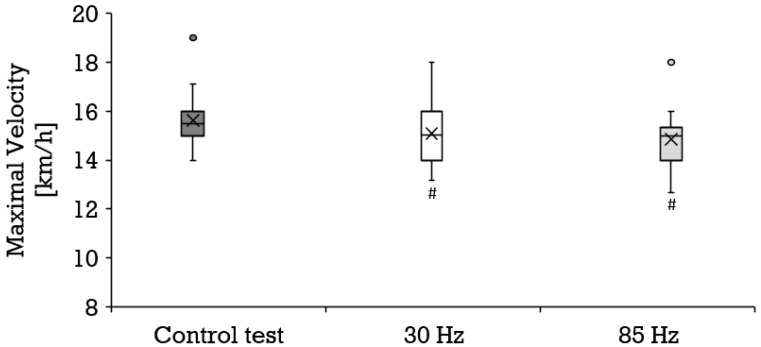
Distribution of the maximal velocity (km/h) depicted as boxplot during the control test (dark gray box, *n* = 22), running with 30 Hz (white box, *n* = 22) and running with 85 Hz (bright grey box, *n* = 22). Each filled circle is representing a discordant value. Significances (*p* < 0.05) compared to the control test are labeled with #.

Compared to the control condition (mean ± SD, 15.6 ± 1.1 km/h), a significantly lower maximal velocity was achieved at both 30 Hz (mean ± SD, 15.1 ± 1.2 km/h; *p* = 0.002; r = 0.65) and 85 Hz (mean ± SD, 14.9 ± 1.1 km/h; *p* = 0.000; r = 0.80) as shown in Figure 5. All three tests engendered a similar maximal oxygen uptake (Figure 2) and perceived exertion (Figure 3). The maximal respiratory exchange ratio was also similar in all tests. The maximal lactate values differed significantly among the three test conditions (Figure 4). Lactate values in the control test (mean ± SD, 12.9 ± 2.4 mmol/L) were higher than during 30 Hz (mean ± SD, 12.2 ± 2.5 mmol/L; *p* = 0.046; r = 0.44) and 85 Hz (mean ± SD, 10.6 ± 2.8 mmol/L; *p* = 0.001; r = 0.83). The greatest difference occurred between the control test (mean ± SD, 13.0 ± 2.4 mmol/L) and 85 Hz (mean ± SD, 10.7 ± 2.7 mmol/L *p* = 0.000). Lactate during the cool-down period after 85 Hz stimulation (mean ± SD, 8.7 ± 3.2 to 10.4 ± 2.6 mmol/L) was significantly lower than after the control test (mean ± SD, 10.7 ± 3.1 to 12.7 ± 2.4 mmol/L) at all sampling points (*p* = 0.000 to 0.001; r = 0.74–0.83). Likewise, 85 Hz stimulation (mean ± SD, 8.5 ± 3.2 to 10.3 ± 2.7) elicited significantly lower (*p* = 0.000 to 0.003) lactate values during the recovery period than stimulation with 30 Hz (mean ± SD, 10.3 ± 2.9 to 12.0 ± 2.5 mmol/L; r = 0.64–0.77).

## 4. Discussion

### 4.1. Overview

The application of superimposed EMS reduced the maximal achieved velocity during an incremental running test on the treadmill. Furthermore, EMS induced a higher oxygen uptake and increased the respiratory exchange ratio during some stages. There appeared to be a conclusive effect on the perceived exertion elicited by EMS independent of stimulation frequency, as results show that running with superimposed EMS led to higher values at the same velocities. With EMS application, the maximal lactate accumulation was lower and 85 Hz resulted in a lower recovery lactate level.

### 4.2. Maximal Velocity

The reduced maximal performance is in accordance with findings of Wahl et al. [12], since exercise terminated earlier when EMS was superimposed on cycling, which was more pronounced at the higher frequency. It can be assumed that neither lactate anions nor hydrogen ions are primarily responsible for fatigue, but supposedly affect performance [29]. Nevertheless, lower maximal lactate levels were measured during both test conditions with superimposed EMS. Other reasons why the maximal velocity was lower during superimposed running have to be considered. Various ions (particularly potassium ions) seem to be involved in the fatigue process [30]. Skeletal muscles show different activation patterns on voluntary and electrically evoked contractions. EMS causes a nonselective, synchronous, and spatially fixed recruitment of motor units. Hence, fast twitch fibers can be activated even at low workloads. The increase in the firing frequency of already recruited motor units and the activation of other motor units as measures against fatigue represent physiological responses during voluntary activities. The higher fatigue accompanying EMS might be related to the fixed recruitment and firing frequency [1]. Furthermore, the 85 Hz stimulation applied in our study represents a higher frequency compared to the naturally occurring frequency in muscles. A stimulation with a high frequency can led to an elevated fatigue [1].

### 4.3. Aerobic Metabolism

Measuring oxygen uptake is necessary to assess aerobic metabolism and can be used to ascertain energy expenditure. The higher submaximal oxygen uptake during treadmill running with superimposed EMS might be attributable to stimulation occurring during muscle relaxation phases due to the impulse interval duration of 10 s and to additional fiber recruitment. The similar maximal aerobic capability during running with and without superimposed EMS in our study is attributable to the requirement of exhaustion during all three tests. However, an even higher oxygen uptake during the stages can be expected by the application of WB-EMS. At least by tendency our results are in accordance with findings in the literature. Banerjee et al. [31] detected a dose-dependent increase in oxygen uptake by 0.7 L/min by applying 4 Hz EMS at 40% stimulation intensity in a supine position without additional loading. Masayuki et al. [13] combined moderate cycling with eccentric contractions evoked by 40 Hz (5000 Hz modulated) EMS. Compared to regular cycling, this resulted in a 2.1 mL·min^−1^·kg^−1^ (average 21%) higher oxygen uptake. Watanabe et al. [15] conducted a continuous cycle ergometer test at 80% of the ventilatory threshold which was characterized by two periods without and two periods with EMS at 4 Hz in an alternating design. The oxygen uptake during both stimulation bouts was higher than during the initial exertion without stimulation. Mathes et al. [18] revealed an acute 7% rise in percentage of peak oxygen uptake during low-intensity cycling with 80 Hz superimposed EMS compared to regular cycling. During exercise without additional weights, aerobic metabolism was intensified by using a 20 Hz WB-EMS shown through a 1.0 Met higher oxygen uptake [5]. The examination of energy expenditure is based on gas sampling when using indirect calorimetry. A higher expenditure was seen during resistance exercise without weights, including squats combined with exercises, when superimposed by 85 Hz WB-EMS [8]. Although not directly comparable, 85 Hz stimulation did not significantly increase oxygen uptake during loaded squats (according to 10 RM) when compared to a control group without EMS application [6].

### 4.4. Anaerobic Metabolism

An increase in lactate oxidation may be involved in the increase in oxygen uptake. [18]. However, the time for steady oxygen uptake to be achieved is prolonged beyond the anaerobic threshold [32]. Therefore, the response could be underestimated. In our study, there were no differences in lactate levels, which represents anaerobic metabolism, during running stages with EMS. In addition, maximal lactate accumulation, as well as values during the cool-down period were lower, though higher values could be assumed considering previous studies dealt with (superimposed) EMS. Considering the specific recruitment pattern of EMS, the involvement of fast twitch fibers even during lower workloads is presumable. At 60 Hz, EMS increased the lactate levels evoked during cycling at 70% peak power output [14], as well as after cycling with 4 Hz at 80% of the ventilatory threshold [15]. Despite the same EMS protocol and an incremental test design on a cycle ergometer, Wahl et al. [12] demonstrated a higher lactate accumulation at the end. Furthermore, lactate at 75% peak power output appeared considerably higher when cycling was accompanied by EMS. In our study, we did not only examine the exercise period but also the cool-down period when selecting the highest lactate accumulation. Furthermore, we assessed stage results and did not consider percentages of the peak power. Omoto et al. [16] examined the effect of cycling at 40% of the peak oxygen uptake accompanied by stimulating antagonists with 40 Hz (5000 Hz modulated) causing eccentric contractions. The workload for the test with stimulation was adapted in accordance with the previously described enhanced oxygen uptake due to EMS [13]. Furthermore, the load was manipulated to achieve the targeted heart rate. A pronounced rise in lactate was detected in the recovery period after cycling with EMS, but the response did not differ compared to regular cycling. During the training study of Mathes et al. [18], low-intensity cycling with 80 Hz stimulation led to an acute 11% rise in percentage of peak lactate compared to regular cycling. Stimulation with 85 Hz superimposed on loaded squats (according to 10 RM) did not engender a significantly different lactate response when groups were compared [6]. The application of 20 Hz WB-EMS additional to exercise without additional weights caused a higher lactate accumulation after completion. The lowest lactate levels were seen after EMS without combined voluntary contractions. Nevertheless, the respiratory exchange ratio was similar during both applications of EMS and lowest during exercise without stimulation [5]. Wahl et al. [12] not only demonstrated a higher lactate at 100% of the peak power output but also a higher respiratory exchange ratio compared to regular cycling when an identical stimulation protocol was imposed. Despite a higher lactate level during cycling with 4 Hz at 80% of the ventilatory threshold, there was no significant difference in respiratory exchange ratio between interventions [15]. Unused muscles or those not under substantial load are able to absorb lactate transported in the blood [33], whereby oxidative muscles working under stable conditions are particularly suitable [34]. Hence, non-stimulated muscles of the upper body, and those of the lower body during the rest period, supposedly metabolize lactate, which might be one reason for similar stage values during all tests. Since lactate anions and protons have their origins in lactic acid [29] and excess carbon dioxide occurs by proton buffering [35], lactate levels and the respiratory exchange ratio are linked. In the assessment of maximum lactate and respiratory exchange ratio, the lower maximal achieved velocity with superimposed running has to be considered.

### 4.5. Perceived Exertion

The stimulation protocol we used yielded a pronounced perceived exertion at 100% peak power of incremental cycle exercise [12]. Additionally, continuous cycling at 70% peak power output with 60 Hz stimulation was perceived as more strenuous than regular cycling, in accordance with the elevated stress level assessed via cortisol [14]. Furthermore, unloaded squats with additional movement tasks were subjectively intensified by 85 Hz stimulation WB-EMS [8]. Both cycling trials additionally ascertained the perceived physical state. Lower values were seen at different time points after the termination of continuous cycling with EMS. Cycling at 70% peak power with superimposed 60 Hz stimulation caused a higher physical pain, sustained for at least 24 h after termination [17]. Stimulation with 85 Hz superimposed on loaded squats (according to 10 RM) engendered a significantly higher muscle soreness 48 h after the first session than squats without superimposed stimulation. In summary, the response after EMS appears delayed and stronger compared to regular exercise [6].

### 4.6. Limitations

To the best of our knowledge, this is the first time a study evaluated the acute effects of a superimposed EMS during an incremental running treadmill test. However, we also need to acknowledge some limitations. The order of the treadmill tests with EMS was randomized but running without stimulation was declared as a control test at the first day—hence it served to assess the performance capability and to detect potential limitations. During running we avoided increasing the stimulation intensity as described elsewhere to consider the tolerability [8] or to enhance the recruitment [17]. Furthermore, the at least partial heterogeneous performance capabilities have to be considered, as runners with different performance levels might experience a different muscle strain at the same velocity. Therefore, the amount of completed stages differs. A complementary assessment of the true muscle strain would have involved collecting muscle damage parameters (e.g., creatine kinase). In this context, subjects who are more familiarized with the application of EMS might tolerate higher stimulation intensities due to specific physiological adaptations and lower (safety) concerns. Additionally, we applied the EMS only to lower body parts. It can be expected that more pronounced responses might be expected from WB-EMS during running.

### 4.7. Perspectives

Future studies should examine the effects of WB-EMS in conjunction with endurance and interval running to allow comparisons with usual training. Among other things, adaptations to long-distance training should be investigated to determine whether a reduction in running velocity or volume, a combination of endurance and strength training, and the induction of better adaptations at the same velocity or volume are possible. Recruitment of subjects who are familiar with EMS applications might be desirable, as the presence of EMS related neuromuscular adaptations can be assumed.

## 5. Conclusions

Electromyostimulation superimposed on running does not interfere movements and can be considered as a feasible exercise application. With superimposed EMS, regular exercise can be intensified. Coaches and athletes could benefit from the increased training stimulus by reducing running velocity or volume, by combining endurance and strength training, and also by inducing better adaptations while maintaining the same velocity or volume. However, EMS-related reactions that might occur due to training must be considered (e.g., rhabdomyolysis). EMS could be an interesting tool for runners, particularly for those covering extreme distances. Future studies should investigate the effect of WB-EMS superimposed on interval and constant running to enable comparisons to usual exercise training. Recruitment of subjects who are familiar with EMS applications might be desirable, as the presence of EMS related neuromuscular adaptations can be assumed.

## Figures and Tables

**Table 1:** Basic and spiroergometry related properties of the participants ^1^.

Parameters	Mean ± SD [Min–Max]
Age [years]	25.8 ± 3.1 [19–30]
Height [cm]	184.0 ± 8.0 [170.0–196.0]
Body mass [kg]	79.5 ± 7.2 [65.0–97.0]
Body mass index [kg/m^2^]	23.5 ± 1.3 [20.5–25.4]
Maximum oxygen uptake [ml·min^−1^·kg^−1^]	50.4 ± 5.8 [38.7–63.3]
Maximum velocity [km/h]	15.6 ± 1.1 [14.0–19.0]
Maximum lactate [mmol/L]	13.0 ± 2.4 [8.8–18.8]
Maximum respiratory exchange ratio	1.14 ± 0.05 [1.05–1.22]
Maximum perceived exertion via Borg scale	19.6 ± 1.1 [16–20]

^1^ Oxygen uptake and respiratory exchange ratio were recorded in 19 participants and the other parameters in 22 participants.

## Data Availability

The data presented in this study are available on request from the corresponding author. Descriptive data (Table S1) is contained within the Supplementary Material.

## Supplementary Materials

The following supporting information can be downloaded at: https://www.mdpi.com/article/10.3390/biology11040593/s1, Table S1: Results of perceived exertion, oxygen uptake, lactate, and respiratory exchange ratio at the end of 8 km/h, 10 km/h, 12 km/h, and 14 km/h, as well as maximum velocity achieved, maximum running distance, maximum running time, maximum lactate, and lactate during the cool-down period, expressed as mean ± SD.
