# Supplementary material for: Acute Effect of Electromyostimulation Superimposed on Running on Maximal Velocity, Metabolism, and Perceived Exertion
Source: Biology (Basel). 2022 Apr 14;11(4):593. doi: 10.3390/biology11040593 (PMC9028827; doi:10.3390/biology11040593)
Supplement: Supplementary file 1 [file biology-11-00593-s001.zip › biology-1611767-supplementary.pdf]

Table S1: Results of perceived exertion, oxygen uptake, lactate, and respiratory exchange ratio at the end of 8 km/h, 10 km/h, 12 km/h, and 14 km/h, as well as maximum velocity achieved, maximum running distance, maximum running time, maximum lactate, and lactate during the cool-down period, expressed as mean ± SD.

**Parameters**	**n**	**Control test**	**30 Hz**	**85 Hz**
Maximum achieved velocity [km/h]	22	15.6 ± 1.1	15.1 ± 1.2 #	14.9 ± 1.1 #
Maximum running distance [m]	22	2862 ± 485	2645 ± 491	2549 ± 449
Maximum running time [s]	22	866 ± 101	818 ± 107	797 ± 99
Perceived exertion 8 km/h [Borg scale]	22	8.7 ± 1.9	8.8 ± 1.5	9.0 ± 1.8
Perceived exertion 10 km/h [Borg scale]	22	11.2 ± 2.2	11.9 ± 2.0 #	11.9 ± 2.0 #
Perceived exertion 12 km/h [Borg scale]	22	14.1 ± 1.8	15.1 ± 2.0 #	15.0 ± 2.0 #
Perceived exertion 14 km/h [Borg scale]	21	16.7 ± 2.1	17.9 ± 2.1 #	18.0 ± 1.9 #
Perceived exertion maximum [Borg scale]	22	19.6 ± 1.1	19.7 ± 1.1	19.5 ± 1.4
Oxygen uptake 8 km/h [ml·min-1·kg-1]	17	31.7 ± 2.3	32.8 ± 3.4 #	32.8 ± 3.1 #
Oxygen uptake 10 km/h [ml·min-1·kg-1]	20	37.8 ± 2.7	38.5 ± 3.3 #	38.6 ± 3.7
Oxygen uptake 12 km/h [ml·min-1·kg-1]	20	43.2 ± 3.0	43.8 ± 3.5	44.2 ± 3.7
Oxygen uptake 14 km/h [ml·min-1·kg-1]	17	48.3 ± 3.6	48.7 ± 3.3	49.4 ± 3.4 #
Oxygen uptake maximum [ml·min-1·kg-1]	17	51.3 ± 5.3	51.2 ± 4.2	51.9 ± 5.2
Lactate 8 km/h [mmol/l]	21	2.5 ± 0.9	2.5 ± 0.7	2.4 ± 0.8
Lactate 10 km/h [mmol/l]	21	3.4 ± 1.3	3.5 ± 1.3	3.3 ± 1.2
Lactate 12 km/h [mmol/l]	21	4.9 ± 1.8	5.2 ± 1.9	5.0 ± 1.9
Lactate 14 km/h [mmol/l]	20	8.1 ± 3.3	8.4 ± 3.1	8.0 ± 3.4
Lactate maximum [mmol/l]	21	12.9 ± 2.4	12.2 ± 2.5 #	10.6 ± 2.8 #, *
Lactate 1 min recovery [mmol/l]	21	12.6 ± 2.2	11.8 ± 2.4	10.3 ± 2.5 #, *
Lactate 3 min recovery [mmol/l]	21	12.6 ± 2.4	12.0 ± 2.5	10.3 ± 2.7 #, *
Lactate 5 min recovery [mmol/l]	21	12.3 ± 2.6	11.7 ± 2.7	10.1 ± 3.0 #, *
Lactate 10 min recovery [mmol/l]	21	10.6 ± 3.2	10.3 ± 2.9	8.5 ± 3.2 #, *
Respiratory exchange ratio 8 km/h	17	0.88 ± 0.09	0.93 ± 0.06 #	0.93 ± 0.06 #
Respiratory exchange ratio 10 km/h	20	0.95 ± 0.06	0.97 ± 0.06	0.97 ± 0.06
Respiratory exchange ratio 12 km/h	20	1.02 ± 0.06	1.04 ± 0.06	1.03 ± 0.07
Respiratory exchange ratio 14 km/h	17	1.09 ± 0.06	1.12 ± 0.05 #	1.11 ± 0.06 #
Respiratory exchange ratio maximum	17	1.14 ± 0.05	1.16 ± 0.05	1.14 ± 0.05

Significances ($p < 0.05$) compared to the control test are labeled with #, compared to 30 Hz with *.
